# On the Treatment of Stone in the Bladder, by Medical and Mechanical Means

**Published:** 1842-04-01

**Authors:** 


### On the Treatment of Stone in the Bladder, by Medical and Mechanical Means. By *R. Willis*, M.D. of the Royal College of Physicians, Physician to the Royal Infirmary for Children, &c. London, H. Baillière, pp. 183. 1842.

Dr. Willis shews but a dull picture of lithotomy. The brilliancy, he says, of the procedure apparently has dazzled both the operator and spectator, and disastrous consequences in unfortunate cases have always been merged in the temporal redemption that is achieved for the sufferer in those that prove successful. But of all of every age and sex who submit to lithotomy one in seven or eight will inevitably be lost immediately, and among those who are in years when the life of man is most truly precious, one at least in three or four will fall its victim. This is, no doubt, pretty true, discouraging as it may be. But what says Dr. Willis of lithotrity? Nothing flattering. This, he tells us, is beyond all question a rotten staff, which leaned upon by all who suffer from stone will certainly fail five-sixths of the number; nearly one-half will find that it is totally inapplicable to their case; from one-third to one-fourth will fall immediate victims to its determined application; and from one-third to one-fourth will escape to lead miserable lives from diseased bladder, and then to die of diseased kidneys; not more than one in three or four of all who were held favourable subjects will find it a safe, effectual, and final remedy for their disease. The public and professional mind has been singularly abused in regard to the value of lithotrity as a general means of treating stone in the bladder.

Although we believe that the success of lithotrity has been greatly overrated, still we would not venture to lay down its statistics so confidently as Dr. Willis. The data have hardly been acquired for any such positive conclusions.

However, Dr. Willis does not leave us with this gloomy prospect. The following remarks are of a more encouraging description.

"Conviction of these truths has lately led physicians back to the long neglected class of medicines called *Lithontriptics*, and there is already evidence enough extant to satisfy us that these medicines, taken internally and used in the way of injection, have even greater powers than were suspected in the palmy days of their employment some century ago.

But lithontriptics, like all other medicines, have no more than a certain limited range of power; and supposing them to fail, and manual interference to become indispensable that the sufferer from stone may have a chance for his life, have we no resource but lithotomy as the general and lithotrity as the exceptional rule? I believe that we possess an almost unfailing refuge in the operation which I have described under the title of Lithectasy. By this procedure the brilliancy of the operation by which a stone is now generally extracted from the bladder will be gone; but the life of the patient will be safe; and this surely is the end which all who are engaged in the practice of medicine as an art, or who are striving to advance it as a science, have in view. Surgical operations generally have been becoming less frequent and less dangerous, ever since the Revival of Letters in Europe. I believe that by the judicious use of lithontriptic medicines, and the adoption of lithectasy, they may be made both much less frequent and infinitely less dangerous than they have yet been found in reference to the treatment of stone in particular." vi.

The first Chapter of the Work before us is on the REMOVAL OF STONE BY SOLUTION. Dr. Willis mingles in an amusing manner argument and history. The following passage contains, perhaps, the largest infusion of the former.

" The alkalis when first propounded as of sovereign efficacy in calculous complaints, were exhibited either in the state of subcarbonate or pure. This implies administration in a large quantity of fluid; and then the conditions were fulfilled which were necessary to the best effects of these medicines. The concurring testimony of all the best authorities of the times gives us assurance of the signal benefit that was often derived from the use of Stephens's medicines, as well as from those of her immediate successors. Stephens's remedy consisted especially of a mixture of calcined egg-shells and Castile soap, which was always washed down with copious draughts of some simple vegetable infusion or decoction. The lithontriptics of all the medical practitioners of the same period were of the same essential nature. The ingenious Dr. Whytt, who enjoyed an extensive reputation for his treatment of calculous complaints, relied upon about an ounce of Castile soap and two or three pints of lime-water in the course of the twenty-four hours. Dr. Chittick, a less ingenuous man than Whytt, though ingenious enough in his way, and who had a large share of the metropolitan practice in cases of gravel and stone, had a tin vessel of the capacity of two quarts filled with weak veal-broth sent to his house every morning for medicamentation, and this quantity of diluent with some solution of potash added to it, was the dose which each of his patients took during the day.

Now, provided the stomach did not rebel against a course of this kind, we are certain, as chemists and physiologists, that it was calculated to act beneficially in a large proportion of cases of stone; in all cases, to wit, in which calculi of lithic acid, of the lithate of ammonia, and, as recent experience has shown, of the triple or mixed phosphates are contained in the bladder. The oxalate of lime calculus, indeed, is the only one upon which the urinous solution of the neutral carbonate of potash or soda thus brought into contact with it must not have had a decided action in the way of solvent or disintegrator.—There is, perhaps, no fact in therapeutics better established on the basis of experience than the influence of weak alkaline solutions upon the matter composing the generality of urinary concretions; and I cannot therefore but hold it a grave error to suppose that the healthy urine is of itself as powerful a solvent of stone in the bladder as any we possess. The most healthy urine always contains a certain proportion of matter so highly concrescible that it will infallibly be precipitated in the solid form if it but meet with a point on which to rest. Qualities, on the contrary, can readily be communicated to the urine by the use of saline and other medicines, that give it positive solvent or disintegrating powers, which, though not of any great force, are nevertheless quite decided, and have only to be maintained for a sufficient length of time to prove of signal efficacy in the end.

Much good, then, was done during the period that alkaline medicines were administered in this way: some were freed from their calculi entirely, and many more escaped from a life of absolute torture to one of comparative ease; for the alkalis have this most admirable quality in addition to the one they possess as direct solvents of stone, that they allay the irritability of the living tissues with which the foreign body is in contact to such an extent that frequently its presence ceases to be perceived, and the person with a stone in his bladder comes at last to be in little worse plight than another who has nothing of the kind.

The progress of chemistry and pharmacy by and by led to the preparation and prescription of the neutral carbonates and of the bicarbonates instead of the caustic alkalis, as heretofore. In the case of the individual,—the celebrated anatomist Mascagni,—who first directed particular attention to this modification, it was attended with all the benefits of the old system. Mascagni himself recovered completely from two or three severe attacks of gravel which he endured, and finally gained entire immunity from any recurrence of his disease under the use of the bicarbonate of potash. But he used this medicine in the old way, that is, along with plentiful dilution. The alkaline carbonates and bicarbonates, however, are so mild, so totally without destructive action on the living tissues of the body, that they soon came to be administered in no larger a quantity of water than was necessary to get down the dose; and then they certainly lost a considerable portion of their efficacy." 9.

We must confess that this flattering picture of the effects of the alkalies does not agree in all respects with our experience. So far from their always allaying irritability, we have very frequently found them occasion it. The liquor potassæ will do this more than the carbonates or bicarbonates, but all will not uncommonly give rise to frequent micturition and uneasiness about the bladder. Nor can we be surprised at this when we consider the disposition to irritability of the bladder occasioned by neutral or alkaline urine.

After a sly hit at the surgeons Dr. Willis makes these remarks:—" To say nothing of the doubt that has always hung around their efficiency and practical applicability, the medical means we possess of attacking stone in the bladder are very slow in their effects; and all know how difficult it is to manage the generality of patients in cases where perseverance is indispensable, and where the result has to be looked forward to at a distant date. In the present time, too, the generality of practitioners are but indifferently acquainted with urinary pathology,—the subject is one that is little studied; or they have no confidence in lithontriptic medicines. The surgeon, indeed, may be little or no better provided than the physician in the important article of the particular pathological knowledge that is necessary here, but he has sounds, and catheters, and lithontriptors and scalpels at hand, and in these he has unlimited faith." We think that this is a little too smart to be perfectly correct. We conceive that it is hardly accurate to say that urinary pathology is little studied. Nor is the faith of the surgeon in his tools unlimited. Quite the contrary. The dangers of lithotomy have always been admitted, sometimes even exaggerated. It is not that too much faith is extended to the operation, but that little is placed in lithontriptic medicines.

The revival of experiments and hopes on this subject has been due to France. In a short essay, observes our author, on the mineral waters of Vichy, published in one of the volumes of the Annales de Chimie for 1826, M. Darcet called the attention of professional men to the property which these waters have of rendering the urine of the drinker alkaline, and to the advantage that might be taken of this circumstance in the treatment of stone in the bladder. He found that from three to four glasses of this mineral water, which contains about fifteen grains of the bicarbonate of soda per glass, taken in the course of the twenty-four hours, sufficed to maintain the urine in an alkaline state. M. Darcet remarked further, that the urine of those who drank the Vichy water was singularly transparent, though the portion which was secreted during the night was often high coloured; and, contrary to wont, that it even continued limpid after putrefaction had made great progress.

Dr. Charles Petit and M. Patissier followed up the investigations of M. Darcet, and presented cases and considerations on the same side. Dr. Willis observes :—

"It is very generally agreed that fragments of urinary calculi of the lithic acid and lithate of ammonia, and of the mixed and triple phosphates, are speedily reduced in size by solution and disintegration when exposed to the action of the Vichy water out of the body; and it seems also undeniable that several persons in whose bladders the presence of calculi had been ascertained by searching, had them either notably reduced in size, or got rid of them entirely, all the symptoms of stone being at the same time relieved or removed whilst using this water internally.

Much about the time that Dr. Petit appeared as the advocate of the waters of Vichy, M. Robiquet, in a paper read before the Royal Academy of Medicine of Paris, (1836,) adduced several instances of the successful exhibition of the bicarbonates of potash and soda, aided by plentiful dilution, in cases of stone in the bladder, patients finally passing the kernels of calculi which had formerly been too large to enter the neck of the bladder, and to get rid of which the operation of lithotomy had been proposed.

The range of cases in which the alkaline carbonates were found to be useful was also greatly extended by the experiments of these various inquirers, particularly of Dr. Petit and M. Chevallier. It had long been generally allowed that weak solutions of the vegetable and mineral alkalis in the state of carbonate exerted more or less of a solvent effect upon calculi of the lithic acid; but it was hardly suspected that these substances had fully as much power over concretions of the phosphates, not, however, in the way of solvents, but of disintegrators, the alkali seizing upon the animal matter, which is a principal bond of union in the great majority of urinary calculi, and the particles of earthy salt then separating and subsiding in the shape of an amorphous powder. M. Darcet, in the course of his experiments, found that even so solid a substance as a *bone*, exposed for a length of time to a solution of bicarbonate of soda in distilled water, was at last completely disintegrated; a solution of gelatin,—a gelatinate of soda,—composing the supernatant liquor, the earthy matter—phosphate mixed with a little carbonate of lime,—forming the powdery precipitate which lay at the bottom of the vessel. Dr. Petit found that calculi of the mixed and triple phosphates, exposed to the action of the Vichy water, actually suffered in many instances a more rapid loss of weight than those of lithic acid or the lithates." 15.

Much opposition and altercation seem to have been going on in Paris between the lithotritists and the stone-dissolvers. The quarrel may be a pretty one, but we need not be a party to it. The Royal Academy of Medicine issued a commission to examine into the operation of the Vichy Waters. They presented their report, from which the following conclusions are taken:—" 1st. Urinary concretions are attacked by the urine when it has been rendered alkaline by the use of the Vichy water taken internally and used in the way of bath; 2nd. It has not been proved that urinary concretions of such a size as to constitute proper calculi, have been entirely removed by these waters; 3rd. Such a removal is nowise impossible; there is even considerable likelihood of its being accomplished; 4th. The question can only be decided by experiment; 5th. The experiment does not seem to present any danger. The committee therefore request the minister of the public works, &c. to accede to the demand of M. Petit," which was to the effect that he might have a certain number of patients affected with stone in the bladder confided to his care at Vichy, with a view to decide the question as to the power or impotency of the natural alkaline waters in this disease.

Dr. Willis quotes nine cases more or less favourable to the action of the Vichy waters. The ninth is the most striking, and we shall confine ourselves to it.

*Case.*—D. B. Jacob, aged 53, entered the Hôpital Beaujon, on the 28th of August, 1838, labouring under stone in the bladder, which had tortured him for several years. A fortnight after his admission, lithotrity was attempted; the stone was seized several times, and splinters were detached, for many pieces were voided on the day of the operation; but acute inflammation of the bladder having supervened, no second operation could be attempted. Even on the 20th of October the patient was extremely ill, complaining of great weakness, of inappetence, and of a fixed and severe pain in the hypogastric region. The excretion of the urine was frequent and painful, and the fluid deposited a great quantity of purulent and glairy matter.

On the 20th of October the patient was put upon the use of the Vichy water in the dose of from a bottle and a half to two bottles a day; almost immediately the urine became less turbid, the appetite and the strength revived, and by the 5th of December he felt himself so much better that he insisted on quitting the hospital.

On the 8th of March the patient returned to the Hôpital Beaujon with a renewal of his calculous symptoms. On the bladder being searched a stone was readily struck. He was ordered the Vichy water for the second time; but none could be had before the 18th. The course once began, however, was continued to the 18th of June, the date at which Jacob was visited by the members of a committee of the Royal Academy of Medicine, for the purpose of being examined previously to his being sent to Vichy at the public expense. The stone in the bladder was caught nine different times by means of a lithometer; the greatest diameter ascertained was seventeen lines and a half, the smallest seven lines (rather more than one inch English in length, by something more than half an inch English in breadth or thickness.)

Placed under the care of Dr. Petit at Vichy in the season of 1839, the patient proved refractory, and only followed the treatment prescribed for him very irregularly. Nevertheless, when he was examined on the 30th of September by the committee, the longest ascertained diameter of the stone was but fourteen lines; the smallest eight lines and a half.

Previously to being remanded in the season of 1840, the stone in this patient's bladder was repeatedly seized by a committee consisting of Messrs. Civiale, Blandin, and Berard, and ascertained to be of the diameters of 13, 14, and 15 lines. The treatment by the Vichy waters, begun on the 23d of June, 1840, was pursued with great regularity to the middle of September, the patient taking from 12 to 25 glasses of the water and a bath daily, and in addition having a stream of the water sent through his bladder by means of a double-current catheter, once, twice, and even thrice a day for some considerable time. In the beginning of August the patient began to pass fragments of his stone, and at the same time he obtained complete relief from his sufferings. On the 18th of September he was sent back to Paris, where, having been sounded on two different occasions by the several members of the committee, it was formally declared that there was no longer any stone in the bladder.

It must be admitted that this is a strong case. Still some fallacy is possible, for the history of stone in the bladder contains cases in which the cure has appeared to be complete, and yet the stone has been present. Sounding is not always a conclusive operation.

Dr. Willis next argues the question chemically. Two objections are constantly raised to the use of alkaline or any other kind of internal medicine in cases of calculus; first, that much valuable time is lost in their administration; and second, that the alkalies especially, far from dissolving or disintegrating urinary calculi, tend rather to cause precipitation from the urine, or at most to change one diathesis into another.

But, says our author, the first objection is founded on the presumed inefficacy of internal remedies generally in cases of stone. And this he disputes.

"The second objection, that the alkalis, far from dissolving urinary concretions, tend rather to cause precipitation from the urine, and to change one calculous diathesis into another, is one that is now of ancient date, that originated in a groundless assumption, and that has been answered over and over again. Urinary calculi of the lithic acid, the most common calculi of all, exposed to the contact of a solution of an alkaline carbonate, or bicarbonate, however weak, are necessarily and in the nature of things acted upon and decompounded. A new salt is formed of sparing solubility, indeed, but still soluble, and the animal matter which formed a principal bond of union between the integral particles of the calculus being greedily abstracted by the alkali, these particles fall away from one another, and the stone becomes powder.

As to the change of diathesis, this too is a mere assumption; nay it is more, it is an error; the alkaline mineral waters and attenuated solutions of the fixed alkaline carbonates have no such power as to cause a deposition from the urine of either the phosphate or carbonate of lime, of the urate of soda, or of the triple phosphate of magnesia and ammonia. The urine of every one who makes habitual use of the fixed alkaline carbonates even in large quantity and sufficiently diluted, is remarkable for its brightness and limpidity. The urine of the female whose case Dr. Bostock has recorded, when taking *two ounces and a half of subcarbonate of soda daily*, was pale and perfectly clear and limpid; and the urine of the drinkers of the Vichy water has been universally remarked for its transparency, a quality which, unlike other urine, it preserves even after it has become highly putrid. More than all this, calculi of the phosphates are attacked by solutions of the fixed alkaline bicarbonates whether prepared by art or presented to us in mineral springs, at least as vigorously as those of the lithic acid." 35.

Dr. Willis goes on to remark, that the bicarbonate of soda in solution, or as it occurs in the Vichy water, is of service in cases of calculus by rendering the urine alkaline. But the alkaline carbonates, whether of soda or potash, are not the only nor perchance the best salts that may be employed in this direction. The neutral citrate, malate, and even tartrate of potash and of soda taken by the mouth render the urine very promptly alkaline, and have precisely the same effect as the carbonate and bicarbonate of the same bases. These neutral salts are in fact decompounded in their transit through the body, making their appearance in the urine in the shape of carbonates, and attacking urinary concretions contained in the bladder just as the bicarbonates do when administered directly by the mouth. These neutral salts, composed of a vegetable acid and an alkaline base, have this advantage over the bicarbonates, that they do not combine with and render inefficient the natural acid of the stomach, which we now know plays so important a part in the function of digestion; they can therefore be taken for even longer periods than the bicarbonates without weakening the stomach, and without interfering with the changes which the food must undergo in that viscus to be made fit to furnish the body with materials for its growth and nourishment.

Dr. Willis alludes to the borates of potass and soda, but doubts if they exert the same power on the animal matter of calculi as the carbonates. He alludes also to the benzoic acid. He has been assured by Mr. Ure that the uro-benzoic acid, which is found in the urine when benzoic acid is taken, has the power of combining with the earthy phosphates, and forming uro-benzoates of most easy solubility. "By treating," says he, "a small calculus of uric acid alternately with a solution of biborate of soda and of benzoate of ammonia, I certainly found it reduced, dissolved, and disintegrated with singular rapidity; and there can be no doubt of the immediate effect which the benzoic acid and the benzoate of ammonia taken into the stomach have in rendering the urine clear and limpid in cases where it had long been turbid in consequence of depositing the lithic acid and lithate of ammonia. M. Leroy, of Paris, is reported to have used these substances with complete success in a case in which renal calculi of the lithic acid had for a long time been formed in large quantities. I believe them fully competent to prevent the formation of every modification of lithic acid gravel and calculus."

*Solution of Stone by Injections into the Bladder.*—Dr. Willis has got together several successful, or seemingly successful cases of this sort. One of them he cites as never having been hitherto quoted.

*Case.*—The patient was a gentleman about 40, in whom symptoms of stone in the bladder and the sufferings that accompany it had been gradually increasing in severity for some considerable time. At length they gained such a degree of intensity that the operation of lithotomy was resolved on; but this having been delayed by the engagements of the surgeon in attendance, Dr. Ritter of Cassel proposed to the patient to try the effect of some of the chemical means that had then recently been propounded by Fourcroy for the solution of the stone. The patient gladly assented, and was forthwith directed on going to bed to take a glassful of a solution of caustic potash in water, of such strength as just excited a feeling of warmth in the mouth, and in the morning, after having made water, to have three ounces of the same solution warmed to 98° Far. thrown by means of a catheter into the bladder. After the lapse of an hour and a half, the bladder was emptied. The discharged fluid was found turbid, and seemed to have lost all its alkaline properties; it let fall a considerable deposit of mucus, and on the surface of this a thin stratum of a grayish or yellowish pulverulent precipitate. The injection was repeated in the evening, when another glassful of the alkaline solution was taken by the mouth.

In a few days the strength of the injection was increased; and the patient was in addition placed in a warm alkaline bath every morning for three quarters of an hour. Towards the eighth day of the treatment decided signs of improvement in the patient's state were manifest. The precipitate from the injected fluid, on its being returned, became more and more copious, and without being absolutely sandy, it was still rough to the touch, and had here and there little masses of a bright yellow or reddish colour interspersed through it. In the same proportion as the precipitate increased did all the symptoms of stone diminish. On the 38th day of the treatment, the pulverulent deposit from the injected fluid having ceased entirely, and the patient complaining no longer of pain or uneasiness of any kind, but feeling himself perfectly well, everything was discontinued. The whole of the precipitate collected together weighed two and a half loths, or about one ounce and a quarter avoirdupois. The patient was seen by Dr. Ritter several years afterwards in perfect health, never having had any return of calculous complaint.

Dr. Willis is sanguine on the subject of injections. He seems to anticipate obstacles from the surgeons.

"I see many difficulties, however, in the way of an extensive continued and fair trial of injections for the cure of stone in the bladder. Surgeons, for example, are disinclined to lend themselves to any means for the cure of stone that do not involve an operation; their education and their habits do not generally fit them to appreciate or incline them to confide in chemical remedies; distrusting the powers of these remedies, they reject them untried; they have a means at command in which they place implicit confidence, and they therefore and naturally give this the preference. Physicians again, in the present conventional division of professional labour, have little opportunity to try any means of removing stone; they are seldom consulted independently in stone cases; and then the physician who meddles with anything *mechanical*—and there is no moving in the treatment of calculus without sounds, &c.—is held as going beyond his province, and is immediately looked upon with jealous eyes, first by his own fraternity, and then by his near kindred the surgeons; so that timidity and the wish to stand well with all, hinder him from profiting by such occasions as he does encounter." 46.

We don't think that this is quite fair to the surgeons. There may be some who look askance at everything but operations. But there are many who cannot be accused of a fondness for operating. *They*, at all events, are not likely to object to a remedy that dispenses with the knife. Nor do we believe that the education of surgeons is so unchemical as Dr. Willis considers it. They know quite enough of chemistry to appreciate the action of a solvent, and are quite liberal enough to hail its employment with pleasure, if it really *is* calculated to be serviceable. But the well-educated surgeons are cautious and deliberate, and have seen the rise and fall of too many methods, to be charmed out of their prudence by the resuscitation of an old one, or the mere birth of a fresh.

In an Appendix to this chapter, Dr. Willis makes remarks on the composition of injections. He prefers the fixed alkalis. The caustic potass, properly diluted, may be employed. But solutions of the carbonates or bicarbonates of the fixed alkalis are, however, to be more generally recommended. Their action, if less rapid than that of the caustic alkalis, is of the same kind, and equally certain. Had we a calculus of pure lithic acid to deal with, a solution of borate of soda or borate of potash would be the best solvent; and this, even in the state of saturation, is not felt as irritating in the slightest degree by the mucous membranes of the body. There is, however, no advantage in employing strong solutions of the carbonates or borates of the alkalis as injections; from three to five grains of the salt to the ounce of water is strong enough.

Among acid injections the best are those that are made with the lactic and nitric acids. According to Dr. Willis, the best of all the methods of using injections into the bladder for the solution of stone is to have a reservoir for the fluid, raised a foot or two above the bed or sofa on which the patient is laid, and to connect this by means of a flexible tube guarded by a stop-cock, with a double-current catheter. In this way, a constant stream of the injection can be kept circulating through the bladder and acting on the stone to the very best advantage. The reservoir should be a double vessel of tinned iron, the outer one being filled with water at from 95° to 98° F. and kept at this temperature by means of a small spirit lamp. There is no necessity for any very rapid current through the bladder.

Calculi of the oxalate of lime must be removed by mechanical means.

The Second Chapter is on the Removal of entire Calculi through the Urethra.

The Third is on Lithotrity. From this we are tempted to extract a rather amusing sketch of M. Civiale.

"To me indeed M. Civiale appears to be one of those favoured children of fortune for whom, to use a vulgar phrase, the bowls always run aright; one of those who without speed is nevertheless foremost in the race, who wins the victory or ere the battle be joined. M. Amussat charges a stone in the bladder of a dead subject; his instrument fails him at the very onset, it goes to pieces, and he is disarmed and retires from the struggle. M. Leroy succeeds him, and makes a hole or two in the stone; but it is a tedious business; all present get tired, and the instrument is at length withdrawn, the stone being left behind but little the worse for the encounter. He perseveres, however, he sees the defects of his apparatus; he has the ingenuity necessary to remedy them, and is effectually implemented at length; but he finds no opportunity to try his fortune on the living body for something like a year, and when the opportunity does at last arrive, the circumstances are unfavourable and he fails.

How different the course of the man on whose birth the genius of good luck has waited! He publishes a machine inferior in point of conception to every other already contrived, imperfect to such a degree that it could never have been used; still he proclaims himself ready to take the field, and scarcely has he done so when he finds not one but two, nay three occasions as 'happy prologues to the swelling act' of his final greatness. His own instrument would not have served him indeed, but what signified this? M. Leroy had been sent before by Providence to provide him tools, and to work he went. Of course he succeeded—succeeded signally! In the first case which he encountered the patient was delivered of his stone in two sittings (Jan. 13th, 1824); in the second case (Feb. 4th) the stone was reduced to powder in four sittings; in the third case (March 4th) it was destroyed with the same ease and expedition.

It was on the strength of these triumphs that Civiale came before the Royal Academy of Sciences, and it was with his mind's eye still dazzled with their glance that Percy drew up the report to which allusion has been made, in which infinitely more than was his due is awarded to Civiale, and far less than belonged to him by indefeasible right is given to M. Leroy. Civiale had indeed the good luck to find the opportunity of performing the operation on the living subject first; but he contributed nothing to the means by which he triumphed. His boasting in regard to lithotrity must be viewed as of a piece with that of the poor furnace-man, who lights the fire and turns on the steam, and then arrogates to himself the beautiful mechanism of the steam-engine and all the wonders it performs." 90.

Passing over Dr. Willis's history of lithotrity, which is entertaining, we shall confine ourselves to the statistics of the operation, a subject of great consequence and of equal difficulty.

We all know the confidence with which lithotrity was promulgated as a safe, a speedy, and a nearly certain cure for stone. It was heralded as a painless substitute for a most painful operation, and as one of the greatest boons that had ever been conferred on suffering humanity. We did not scruple at the time to express our disbelief, and we noticed some cases which far from supported the statements of the lithotritists. We stood almost alone in our scepticism, and the bulk of the profession gave itself up to delusive expectations of a success which has never been attained. This the data we shall immediately refer to will establish.

Before we proceed to them we would make one observation. Whatever may be a man's honour and veracity, experience would seem to shew a singular discrepancy between his own account of his own doings and that supplied by other people. Without attempting to explain this, it may be admitted as a fact, that medical men would appear not to rise superior to the remainder of the world, nor to claim for their reputed successes a smaller quantity of faith than their neighbours. It is singular, then, what a difference obtains in the records of private and of hospital practice. Could we trust the parties, the former is the El Dorado of medicine, where desperate cures are constantly accomplished, and a marvellous uniformity of success obtains. This will appear sufficiently in the sequel, if our readers at present entertain any doubts of it.

M. Civiale has had a large practice and a wide experience, nor has he declined to publish the results. In his Traité de l'Affection Calculeuse, p. 613, he speaks of the number of persons affected with calculus who had sought his assistance up to the year 1836. They amounted to 506. Of these 199 were either unfit subjects for lithotrity, or were otherwise prevented from submitting to the operation. Supposing the whole of the 199 to have been really unfit, this would be in the proportion of one in two and a half very nearly, to whom lithotrity held out no chance of relief, or who, were they subjected to the operation, would almost certainly lose their lives. We have, therefore, 307 subjects favourable for the operation. Of these, says M. Civiale, (op. cit. p. 630,) 296 were completely cured; 7 died; 3 were only partially relieved; and in 1 the issue is not known.

This looks well, but some ill-natured persons ventured to doubt if M. Civiale was *quite* so lucky, and they set about inquiring into his cases very pertinaciously.

In a report presented by Barons Larrey and Boyer to the Royal Academy of Sciences in the month of April, 1831, upon a *compte-rendu* or statement in regard to the patients affected with calculus confided to the care of M. Civiale at the Hôpital Necker, we find it stated with something like a censure that M. Civiale should have confined himself to the mention of five cases in which he had recurred to lithotrity with a success more or less decided; but passed by in silence the patients who underwent the operation of lithotomy (after having been vainly essayed by lithotrity), so that, say the Reporters, " we should have remained in complete ignorance of the fate of these individuals had we not seen the *movement* of the Hospital, which M. the Controller was obliging enough to lay before us. We find," continue the Reporters, " that twenty-four patients (not sixteen, as stated in M. Civiale's compte-rendu) had undergone the operation of lithotrity or lithotomy. Of these twenty-four patients, of whom *six* were cut, [after lithotrity had been essayed in vain,] *eleven* died more or less immediately after the operation."

M. Civiale would seem to play Falstaff backwards—for his men in buckram grow few instead of many. Here then are eleven out of the seven who died.

" Let us go on," says Dr. Willis, " to public document the second. This is a report to the Royal Academy of Sciences presented by Messrs. Boyer, Double, and Larrey, upon the operations performed at the Hôpital Necker during the years 1831 and 1832. ' Fifty-three patients affected with calculus were received at the hospital. Of this number twenty-seven treated by lithotrity were discharged completely cured; sixteen having had various attempts at lithotrity made upon them, the operation was definitely found impossible, or useless, or it proved fatal. Of these sixteen ten died and six remained unrelieved. Eight other patients were subjected to attempts at lithotrity and then to lithotomy; of these five died and three recovered.' If we analyze this statement we find fifty-three receptions and twenty-seven recoveries; which is as nearly as possible one recovery in two cases by means of lithotrity; but as three recovered by means of lithotomy after the failure of lithotrity, we have three to add to the list of cures, which therefore amount to thirty in all. On the other side we find ten deaths as immediate consequences of lithotrity, and five more after lithotrity and lithotomy combined, that is, fifteen deaths in all. Eight cases remain unrelieved, which must terminate fatally within a brief period, all the more quickly, for the sounding, &c. which the subjects of them doubtless underwent at the Hôpital Necker. *Fifteen* dead, *eight* unrelieved and expecting death, make *twenty-three* cases of non-success to *twenty-seven* of success by lithotrity." 97.

M. Velpeau has pressed M. Civiale even harder. He has furnished an analysis of five series of M. Civiale's cases. Here is the Table.

| Series. | Number. of Cases. | Cured. | Dead. | Unrelieved, the stone remaining. | Otherwise Success in. | Failure in. |
|---|---|---|---|---|---|---|
| 1st. | 83 | 41 | 39 | 3 | 41 | 42 |
| 2nd. | 24 | 13 | 11 | 0 | 13 | 11 |
| 3rd. | 53 | 30 | 15 | 8 | 30 | 23 |
| 4th. | 30 | 18 | 8 | 4 | 18 | 12 |
| 5th. | 16 | 6 | 7 | 3 | 6 | 10 |
| | 206 | 108 | 80 | 18 | 108 | 98 |

"That is to say, of 206 patients operated on, 108 (a very little more than *one in two*) recover immediately; 80, or nearly *one in two and a half* die; and 18 retain the stone, and will be lost. *One hundred and eight cases cured,* to *ninety-eight in which death is immediately induced* or may not be averted within a brief interval of time. This is a very different tale from the one told by M. Civiale himself; the reader may adopt the conclusions of whichsoever of the two statements he pleases. There can be no question as to the one that conveys the truth.

But M. Civiale can readily be convicted of mis-statement out of his own mouth, or by the act of his own pen; indeed it is from data furnished inadvertently by himself that M. Velpeau has arrived at the conclusions as just given; and whoever will be at the trouble to turn to the work entitled 'Paralele des diverses moyens de traiter les calculeux' will straightway find himself picking his steps over a sort of battle-field. In ten pages of the preface he will meet with a registry of *eight deaths,* and between the 12th and 65th page of the body of the book, no fewer than *fourteen failures* make their appearance,—twice, in a single page, there are records of two disasters." 99.

Dr. Willis proceeds with much charity and good nature to explain, as a trifling slip of memory, these little inaccuracies of M. Civiale.

"All the cases," he observes, "in which the process is essayed, but unsuccessfully, in which one or two or even three attempts have been made to seize and perforate the stone, and even in which it has been seized and perforated once or twice, but in which it is found impossible to proceed in consequence of pain, of inflammation excited in the bladder, &c. &c.—all such cases are thrown out of the account; *in them lithotrity was not performed;* those that die under these circumstances, *die before the operation,* or from some other cause than the operation. Where men are deeply interested, where they think their reputation is at stake, they deceive themselves, and then they try to deceive others. So respectable a surgeon as Mr. Martineau, who prided himself on his success as a lithotomist, thought that he did not select his patients. 'In this number' (eighty-four cases in which he had performed lithotomy), he states that 'no selection of patients was made, as I never rejected any one who was brought for operation' (Med. Chir. Trans. vol. xi. p. 409.) But what is the language of his contemporaries? Let us consult Mr. Crosse: 'During the many years that I witnessed Mr. Martineau's public practice, he carefully selected his patients;' (On Urinary Calculus, p. 155.) Unless he had a great chance of success, therefore, he never ran the risk of failure. In charity we must presume that it is the same with M. Civiale, although candour also compels us to admit that however emphatically warned he will not consent to be set right." 100.

This seems at first sight unaccountable. But the truth is it would be very inconvenient to be set right. The real statistics would not look so well in print, would not attract *good* patients. There's the rub. Dr. Willis quotes a case in point from M. Civiale.

*Case.*—M. Bizouard, aged 65, though he had long suffered from symptoms of stone, had such a dread of every thing in the shape of a surgical operation, that it was only when fairly vanquished by his sufferings that he would consent to have his bladder searched. This being done at length by M. Hervez de Chegoin, a stone was struck. M. Civiale being called in a few days afterwards, ascertained without any difficulty that the bladder contained a foreign body,—of about the size of a large walnut. The urethra was sensibly contracted below the pubic arch, and the prostate was larger than natural. Some preliminary treatment, the necessity for which was indicated by the character of the patient and the irritability of the parts, was then had recourse to. "*An exploration of the bladder made by means of the instruments of lithotrity*," says M. Civiale," "convinced me subsequently that it would be an extremely difficult matter to crush the stone, in consequence of its size, the enlargement of the prostate, the small capacity of the bladder, and the general irritability of the subject. *This perquisition was long and painful. It was followed by a little fever, difficulty in making water, &c.—These accidents never subsided.* Lithotomy was proposed to the patient as his only resource. A consultation was therefore called, and lithotomy was determined on. The day was fixed, the patient had the perinæum shaved, he was laid on his bed, and the sound was passed as the first step in the operation, but no stone could be discovered. It was searched for in vain by more than one experienced hand; the patient had to be put to bed again. Inflammation of the bladder now set in, and the patient died four days afterwards. Nous trouvons là," continues M. Civiale (l. c. p. 378,) "une preuve éclatante que les explorations pratiquées au moyen du cathéter ordinaire peuvent être plus dangereuses que celles pour lesquelles on employe les instruments de lithotritie."

We agree with Dr. Willis that it is rather too bad to lay this death upon the sound. And yet M. Civiale is perhaps right, for as the last drop makes the bucket run over, so the sound settled the business. But before the last drop the bucket must have been full, and the "perquisition" of the bladder made a very complete preparation for the sound. The latter, in fact, was merely the final puff when the flame was flickering.

For a "perquisition," our readers must know, is no joke. Though it is not to be called the operation, it is something so wonderfully like it that the uninitiated cannot distinguish the difference. M. Velpeau's account of it is not bad. "They introduce the litholabe, the lithontriptor or the percutator into the bladder, where its point is moved about to ascertain the existence and the position of the stone. They then open the instrument, its branches are separated to seize or embrace the foreign body, and to appreciate its size and its form. They then endeavour to perforate, to crush, or to fracture the stone by acting on the other and outer end of the instrument, which is thick and straight within the urethra. All this is done once, twice, or three times at intervals of a few days. And now, doubtless, some one will ask, in what the operation properly so called differs from these preliminaries? *Ma foi, je n'en sais rien!*"

It is worth while inquiring what M. Civiale understands by patients who died "without having had any operation performed on them." Such a case was that of Lecomte. The particulars were obtained by M. Velpeau from some private records of the Hôpital Necker, and he dares M. Civiale to dispute them.

"Lecomte had suffered from stone for two years, and had an ample urethra: the intention was to have begun the operation on the 5th of June, 1830. The instrument was passed into the bladder previously injected. Acute pain was experienced in the region of the prostate. Scarcely had the instrument been expanded when the pain became intolerable. The instrument had to be withdrawn before the stone could be attacked. From this time there were incessant calls to micturate, accompanied with tenesmus and excessive pain during the emission of the urine. *Death* on the fifth day afterwards." 103.

It is certainly true that this patient did not fairly undergo the operation of lithotrity. But it is also true that that operation was attempted, and that the attempt was fatal. Is it honest to exclude such a case as this from the statistics of the operation? There can be but one answer—it is *not* honest.

Now let us look at another class of cases in the list of M. Civiale, that of deaths from lithotomy.

"'Godailler, aged 57, had suffered from stone for three years. The instrument was introduced the 17th April, 1830, and the stone charged with the greatest ease; *the drill was worked*. After this operation the calls to urine became extremely frequent; in the evening the patient had a rigor, and then he was attacked with fever. A second *sitting* took place on the 24th; the febrile paroxysm returned with renewed violence; the urine loaded with mucus acquired a sanguinolent tint. The state of the patient getting worse and worse, he was cut (and died.)" 104.

But, according to M. Civiale, he died of lithotomy, not of lithotrity. When such reservations as these are practised, what confidence can be placed in the statements of the man who is proved to be guilty of them? What can we think of M. Civiale's goodly list of cures? No degree of credit can now be attached to them, and we do not envy the position of M. Civiale himself. Sure we are that, in this country, the stain which rests upon his candour would not be esteemed a light one. The moral tone of French society is such that men seem to bear themselves quietly under imputations which would cover them with indelible disgrace here. "Liar," and "rogue," are banded about in the disputes of Parisian surgeons and physicians, as familiarly as household words. And the members of our honourable profession on the other side of the channel appear to receive these flattering epithets with the utmost complaisance.

When we turn from the highly-coloured statements of M. Civiale to the sober statistics of others, we are equally struck with the appearance of truth and the absence of success.

1. M. Velpeau had under his immediate care twelve cases of calculus, in which he performed or attempted to perform lithotrity. In the 1st case, lithotrity had to be abandoned on account of the sufferings of the patient, who remained unrelieved; in the 2nd case the patient was cured; in the 3rd he died; in the 4th lithotrity had to be relinquished, after which, lithotomy was performed; in the 5th, the operation of lithotrity was also found impracticable, and lithotomy was had recourse to; in the 6th the patient was cured; in the 7th he was also cured; in the 8th he died; in the 9th he was cured; in the 10th, he died; in the 11th, he was cured; in the 12th, lithotrity had to be given up, and lithotomy substituted. Of the 12 cases, consequently,

5 were cured by lithotrity, (not one in two);
in 1 the operation was abandoned, and the patient remained unrelieved;
in 3 lithotrity was given up as impracticable, and the patients were cut and recovered;
and 3 died of the operation.

5 in 12 is *one in two and a quarter*, nearly, in which success follows the operation of lithotrity.

4 in 12 is *one in three* in which lithotrity is unavailable,—the operation cannot be performed.

3 in 12 is *one in four* in which a fatal result ensues.

It is difficult, we think, to dissent from the following inference of Dr. Willis. "The effect of the operation gone on with in the four cases in which it was abandoned may easily be conceived; it were not saying too much to maintain that in the hands of a man committed to the operation of grinding or crushing, four deaths more would have been added to the list of the mortality, when we should have had five recoveries counter-balanced by seven deaths."

2. M. Bancal is a warm advocate of the operation, and the author of "A Practical Manual of Lithotrity." Let those, says Dr. Willis, who imagine that lithotrity is a pleasant pastime rather than a most serious business which often brings life into jeopardy, take the trouble to peruse M. Bancal's cases, and then decide between those who would greatly restrict lithotrity and those who would apply it indiscriminately. Among the particulars of these cases he will find mention made of repeated and violent paroxysms of fever, the sequence of severe pain; of retentions of urine; of inflammations of the testes, and bladder, and knee; of treatments protracted through four months, and relief not obtained after all, &c. &c. Among the fourteen cases he will find that in *one* only could the cure be said to be satisfactory and complete; that in *one* after fourteen operations the patient seemed to be delivered of his stone, but was not cured; that in *four* lithotrity was essayed in vain, but the patients were cut and recovered; that in *four* lithotrity having been essayed, the patients remained unrelieved and would perish; and that in *four* death ensued immediately, or within about a year, from the effects of the operation.

3. "'In a grand total of 1003 patients who have come under the hands of the lithotritists,' says M. Velpeau, speaking in 1835, '616 only have been delivered of their calculi: and 387 have died or have not been relieved.' Entering into a few particulars, the same excellent writer informs us elsewhere, that 'the patient operated on by M. Civiale at Florence in 1835, was not cured. A merchant of Lyons, a patient from Anjou, another patient from the country, two patients from the department of the Seine and Oise, the husband of a midwife of Paris, a printer, a patient of M. Roux, two patients of mine, a patient of M. Lenoir, Colonel Rankin an Englishman, another personage of the same country, a patient operated on by Mr. Attenburrow, a patient of Mr. Oldknow, a patient of M. Colliex, a patient of Mr. Norris, and others still, all fell victims to lithotrity. It would therefore, I repeat it, be to abuse the public to hold out this operation as free from everything like danger.'" 107.

4. In the course of the year 1834, says M. Sanson, there were either five or six (certainly not more than six) operations by lithotrity at the Hôpital Necker. *Two of the subjects of these operations died.* Since the first of January of the present year (1835) three cases of stone have been received into the same establishment. One has died of the operation; the second has been alarmingly ill in consequence of it; the third is perfectly well,—but he has not yet been lithotrized.

5. Of 200 cases of lithotrity, says M. Begin, of which M. Civiale has intelligence, performed at Paris, Bordeaux, Nismes, Avignon, London, Edinburgh, Vienna, Munich, Philadelphia, &c. hardly 100 cures are reckoned.

As Dr. Willis observes, the fatal cases reported are immediately fatal. We have no authentic record of those fatal subsequently. Had we that, lithotrity would wear a still less inviting garb.

6. Mr. Fergusson, has related, in the Edinburgh Medical and Surgical Journal, the particulars of seven cases in which lithotrity was performed. Dr. Willis gives an abridged account of them. This we shall omit, and content ourselves with his comment on them.

"In these seven cases, therefore, we see but two recoveries from stone through the means of lithotrity. One man is delivered of his stone indeed, but he is left with a diseased bladder, which is, if possible, a worse evil than the stone. One is dismissed with his stone in fragments still contained in his bladder, and one dies immediately from the effects of the operation. That is to say, we have as good as three deaths in seven cases: for he who escapes from lithotrity with a diseased bladder dies; and he who is unrelieved of stone by one process, and is left beyond the pale of relief by any other, perishes also. Two are cut, lithotrity having failed, and recover. Two recoveries in seven cases, and each of these achieved at the cost of vast suffering to the patient, and certainly with imminent peril to his life!

Besides these seven cases, Mr. Fergusson had cognizance of many others, in which lithotrity had been performed, but which were less immediately under his own eye than those the details of which he has given. He informs us, however, that out of eighteen cases in which he had known lithotrity to be performed, *six were cured; seven were not cured;* and *five died.* Even in the number of reputed cures, too, there is one case in which there are strong reasons for suspecting a *return* (quere, *a continuance) of the disease;* there is a second, in which, though no stone can be felt, 'the patient has suffered almost as much since he was operated on as he did previously to coming under the surgeon's care. Indeed,' continues Mr. Fergusson, '*in two only of these (eighteen) cases can the operation be said to have been attended with that happy success which has been generally claimed for lithotrity.*'" 113.

7. Dr. Willis next examines twelve cases reported by Mr. Key. These we have, on a former occasion, adverted to more particularly, as indeed we have also to the cases of Dr. Fergusson, and we shall merely notice the brief summary of them furnished by our author.

Of the 12 patients 3 were cured by lithotrity, and 3 after vain attempts by this means, were cut and recovered.

6 died, of whom *one* with abscess in the prostate soon after the operation, *four* with protracted sufferings in consequence of fragments remaining in the bladder, and *one* with disease of the bladder brought on or aggravated by the operation, but whether with any fragments left in its cavity or not was not ascertained. We have consequently *one case in four* cured by lithotrity, and *one case in four* cured by lithotomy, lithotrity having failed; finally, we have *one death in two* of the entire number of patients who were subjected to this means, these patients having all or with a single exception been under the hands of one of the most skilful lithotritists of the day; a man who, to his literary and scientific knowledge, the result of a most liberal general and professional education, adds a rare mechanical genius, and a dexterity that was never surpassed.

Those who have witnessed Baron Heurteloup's operations will subscribe unhesitatingly to the above eulogy on his dexterity. But the very fact of that dexterity is the more damnatory of the want of success.

8. Dr. Willis quotes from M. Souberbielle another bill of indictment against lithotrity, made up of these formidable counts.

M. Michali was brought to such a pass by attempts to crush the stone, that when immediate relief became the indispensable condition of present safety, and M. Souberbielle was brought in for the purpose of cutting him this eminent lithotomist saw that the patient was lost, and had to decline interfering; the unfortunate Michali, in fact, died the day after that on which it had been proposed to operate.

M. Turgot, having submitted to lithotrity, had his urethra and rectum perforated; he however recovered from the double infirmity under which he laboured, by means of lithotomy, which Dupuytren performed.

M. Petiet, lithotritised by two different hands, had his urethra torn; five abscesses about the perinæum were the consequence; the patient, sunk in courage and weakened in body, would not submit to any other measure proposed for his relief, and died with his stone in his bladder.

M. Senecal had the urethra and left corpus cavernosum torn in vain attempts to perform lithotrity; whence infiltration of blood, inflammation, &c.

M. Gasselin, treated fruitlessly by lithotrity, had among other accidents an abscess formed between the upper and anterior wall of the bladder and the abdominal parietes.

M. Cornu died in great pain after nineteen operations of lithotrity; his bladder was violently inflamed.

M. P——, after an attempt to crush his calculus, was seized with inflammation of the bladder, and died in the course of a fortnight.

A veterinary surgeon of Puy-de-Dome, at the inspection of whose body M. Breschet was present, was found to have had his bladder pierced as if by a pinking-iron, whence ensued infiltration of urine, gangrene, and death.

A retired officer of the Imperial Guard, the subject of calculus, but in other respects enjoying good health, was treated by lithotrity, from which he suffered dreadfully. He returned to the bosom of his family, where he died some short time afterwards. On a post-mortem examination, the bladder was found lacerated in several places, and in a state of suppuration.

A counsellor in the court of Besançon came to Paris in 1829, to place himself under the care of M. Souberbielle. He was dissuaded from this, and induced to submit to lithotrity. He died during the process; inflammation of the bladder and peritoneum carried him off in the short period of three days.

Baron Fouché submitted himself to the treatment by means of lithotrity; he died of inflammation of the bladder before it was achieved.

A man named Jean died after the operation of grinding the stone, of acute inflammation of the bladder with mucous discharge.

General Roguet, on the first introduction of the lithontriptor, had the urethra lacerated, and an abscess of the scrotum in consequence. He was cut and recovered.

Mr. H. Chaussier, who, in 1834, had been under treatment by lithotrity for nearly a year (!) had twice had the bladder pinched, and two pieces of the mucous membrane torn off by the instruments.

"I could greatly multiply instances," says M. Souberbielle, "but I do not pretend to write the martyrology of lithotrity. I have besides had occasion to perform lithotomy on upwards of thirty patients who had had lithotrity essayed upon them, more than twenty of these having been under the hands of M. Civiale. The whole of these patients assured me that they suffered more from a single sitting of the grinding process than from the operation of lithotomy.

I was present at a lithotritic sitting in the house of M. Blanche, performed on a patient who, after the *thirty-sixth operation and fifteen months of treatment,* was not yet delivered of his stone. I am cognizant of another fact which may seem incredible, and where one does not know which to admire most, the courage of the patient or the courage of the surgeon. *Eighty-four operations* were done in this instance in the course of one year and eight months, and the patient was still undelivered. Worn out by the inutility of his sufferings, the patient ended by desiring to have lithotomy performed; this was undertaken by the same attendant, and with the effect of delivering the sufferer effectually, for his shattered frame could endure no more; he died."

We quite agree with Dr. Willis that, formidable as these cases look, they are after all what we might expect. When we consider the amount of irritation which the mere presence of the calculus excites, the severe symptoms that occasionally ensue from the simple operation of sounding, we cannot surely be surprised that the introduction of the large lithotritic instruments, the hunting for the stone, the seizing and crushing it, the sharp fragments into which it is broken, and the repetition of the processes for its removal, should be fraught with dangerous excitement to a mucous membrane in a state, perhaps, allied to chronic inflammation, and connecting, by its continuity, the kidney with the organ we are treating so roughly.

Dr. Willis observes—The singular increase of irritation that takes place in consequence even of the *spontaneous* breaking up of calculi in the bladder, a phenomenon which sometimes occurs, and the danger to life that ensues thereon, is strikingly illustrated by the circumstances and the issue of a case which is related by Mr. Liston. A medical man, who had laboured under symptoms of stone for a great many years, and who by sounding himself had ascertained the existence of a stone in his bladder ten years previously, was one day met by Mr. Liston in consultation. In three days after this Mr. Liston was summoned to this unfortunate gentleman in a moribund state, from inflammation of the whole urinary system, his urethra being at the same time blocked up by large fragments of stone. "It appeared," says Mr. Liston, "that on parting with me he had been summoned to an urgent case of labour. He ran quickly down a steep street, and at the bottom was seized with an urgent desire to make water, which he did in small quantity mixed with much blood, and passing some pieces of stone with sharp angles. He became alarmingly ill; went on from bad to worse; had retention of urine from obstruction of the urethra; suppression of urine followed, and death terminated his sufferings in a few days. Many portions of the calculus had been voided; but much of it with the nucleus still occupied the bladder and urinary passage. The kidneys were dark-coloured, and one approached to a gangrenous state."

Dr. Willis sums up by deciding on the case in which lithotrity is admissible. "Lithotrity is admissible, and only admissible, in cases in which the bladder is perfectly healthy, and in which the stone is small,—

of the size of a filbert, a shelled almond, or it may be a nutmeg at the utmost; under all other circumstances it ought to be held impracticable. In other words, lithotrity is admissible where it is estimated that the stone can at one sitting be seized and reduced to fragments of sufficient minuteness to be passed by the urethra. No second, certainly no third operation ought ever to be contemplated. *If the patient who has had lithotrity performed upon him is not relieved at once, he is in imminent danger of losing his life.*"

Perhaps this is laying down the law too authoritatively. We have seen patients recover perfectly well who have submitted to two or three sittings. No doubt the proper case for lithotrity is that of a small stone and a healthy bladder. But it would be going too far to affirm that the sitting must never be repeated.

We think we see in what follows the same disposition on the part of Dr. Willis to pronounce more dogmatically than the evidence warrants on the case for operation.

"Lithotrity, however, *ought* to be an operation without much or any pain; when it is not so, the operation is ill-chosen; it ought not to be attempted if it prove painful. If a patient or his attendant have a doubt as to the fitness of the case for treatment by crushing, and the stone be not large, let the amount of pain in all the preliminary steps of the operation guide them both as to the propriety of proceeding with lithotrity or giving it up. Let the instrument be slowly introduced, and the power of the urethra and prostate to bear the presence of the stiff and straight iron rod be tested for a minute or two. If there be no complaint, the business may be proceeded with; if the sense of uneasy distention and forcing increase and by and by amount to pain, let the instrument be forthwith removed, and the hope of relief from lithotrity be at once abandoned. If no pain ensue the instrument will then be gently opened, and an effort made to seize the stone; even in doing this there ought to be little pain; no determined effort to seize the calculus should be permitted; the surgeon who makes such an effort under the circumstance contemplated, is false to the trust reposed in him: if the stone cannot be seized readily and at once without suffering to the patient, lithotrity is to be abandoned, the case is not adapted for treatment by its means." 125.

The only way that we know of to determine satisfactorily a question of this kind, is a reference to facts. Unquestionably, then, we have seen cases succeed perfectly in which the lithotritic process has been very painful. Indeed, to speak the truth, we have never seen the lithotritic process which has not been so.

The fourth Chapter is on LITHOTOMY. Dr. Willis dwells on the importance of a small *incision* and gradual dilatation of the prostate and neck of the bladder. A note on Mr. Martineau is not uninteresting. Mr. Martineau says—

" 'Should the stone be large, or there be any difficulty in the extraction, rather than use much force, while the forceps have a firm hold of the stone, I give the handles to an assistant, who is to draw them outwards and upwards, while the part forming the stricture is cut, which is easily done, as the broad part of the blade becomes a director to the knife; and rather than lacerate, I have often repeated this enlargement of the inner wound two or three times.'

But observe what Mr. Crosse says in regard to one of Mr. Martineau's operations, in which he held the staff for that distinguished surgeon. 'I distinctly felt the knife touch the groove of the staff at the second cut; what was farther done with the scalpel was soon accomplished; but I felt convinced *that the prostate gland was only partially divided, and the neck of the bladder not incised;* still the blunt gorget being very conical entered readily; and the staff withdrawn, the operator introduced his fore-finger upon the concavity of the gorget into the bladder, *forcibly dilating its neck,* and touching the stone. After the forceps had grasped the stone, the scalpel was twice employed to enlarge the wound downwards and outwards......... still the stone, weighing an ounce and three quarters, *was not extracted until great force had been applied.*'

This case proved unsuccessful. On inspecting the body of the patient, Mr. Crosse found the prostate divided to above two thirds of its depth, in a direction backwards and a little outwards. The neck of the bladder was not lacerated, so it must have yielded to allow the large calculus to be extracted, as neither the neck of the bladder nor the posterior portion,—nearly one third,—of the prostate gland, had been divided in the operation.

In connexion with the same case, Mr. Crosse informs us that, after the blunt gorget was introduced and the staff was withdrawn, Mr. Martineau 'was accustomed to introduce his left forefinger (which was particularly long and large) upon the concavity of the gorget into the bladder, *forcibly dilating the opening and using the finger as a powerful but safe instrument for rendering the neck of the bladder ample to admit the forceps. The force and determination with which the finger was thus used, dilating if not lacerating the remaining undivided portion of the prostate gland and neck of the bladder, I always regarded as a peculiar and intrinsic part of Mr. Martineau's method of operating.*' Mr. Martineau's own description of his operation gives us the idea that he cut every thing which opposed resistance to the issue of the stone. We see that he, in fact, *cut very little and took out the stone by main force,* as all successful lithotomists have done.

I have thought it of great importance to quote this explanation of Mr. Martineau's operation, because an erroneous impression of its nature is all but universal, and has I believe proved highly detrimental to the success of lithotomy in the generality of hands. Dr. Marcet, in his widely circulated work on Calculous Disorders, (1816,) first gave it publicity. He says, (Appendix, p. 193,) 'Mr. Martineau determined to lay aside the cutting gorget *and trust to making a large opening into the bladder with the knife.*' But whoever makes free internal incisions will very certainly not perform lithotomy with the success of Mr. Martineau. We are indebted to Mr. Crosse for telling us what this distinguished surgeon's operation was in fact.

Mr. Crosse himself, after describing the particulars of a case in which, the blunt gorget not having previously been got through the prostatic urethra, 'the forceps were at last, by main force, made to enter the bladder,' and in which 'immense force was used in the extraction of the stone,' states, that he had 'repeatedly known instances of the scalpel not being carried deep enough, and of the prostate gland in consequence being pushed before the blunt gorget, the operator succeeding at length [in getting into the bladder] by *dilating* the prostatic urethra and neck of the bladder; and the result of such cases has brought conviction to my mind that this error is less dangerous than cutting too freely the prostate gland and neck of the bladder. If the surgeon misuse a keen scalpel or cutting gorget, the effects are generally fatal to the patient." 133.

The statistics of lithotomy form a prominent object of attention on the part of Dr. Willis. He has, with great diligence, collected and compared a number of published Tables, to the most important of which we shall refer.

1. A very valuable summary of the operations performed in the hospital of Norwich or its neighbourhood is furnished by Mr. Crosse. It presents us with a bird's-eye view of 704 cases. Of these, 387, considerably more than one half, were children and young persons under 20 years of age, and 35 were women. But here is the Table.

| Ages. | Number of Cases. | Number Cured. | Number Dead. |
|---|---|---|---|
| YOUNG PERSONS | | | |
| From 1 to 10 | 281 | 262 | 19 |
| —— 11 to 20 | 106 | 97 | 9 |
| ADULTS. | | | |
| —— 21 to 40 | 96 | 88 | 8 |
| —— 41 to 60 | 147 | 112 | 35 |
| —— 60 to 80 | 78 | 56 | 22 |

In the first series the mortality is extremely small, and even in the second it still continues wonderfully low, amounting in the former to 1 in $14\frac{5}{6}$ths nearly, and in the latter to about 1 in $11\frac{2}{3}$rds. Up to the age of puberty, the operation for stone by cutting, therefore, appears to be attended with comparatively little danger. Even in the third series of cases—age from 20 to 40—the mortality is little increased; it amounts to 1 in 11 exactly. In the fourth series—age from 40 to 60,—the mortality rises rapidly; it amounts to 1 in $4\frac{1}{5}$th very nearly, and in the fifth series, it is still higher, being about 1 in $3\frac{3}{4}$ths. The mortality, according to the Norwich data, up to the age of puberty, is about 1 in 12; after 40 it is in the proportion of somewhat more than 1 in 4. The mortality from lithotomy at all ages is in the proportion of 1 to $7\frac{32}{91}$.

2. In Austria, of 197 cases of calculus which presented themselves, 138 were selected as favourable for operation, and 59 were rejected as past relief by this means. Of the 138 who were operated on, 105 recovered and 25 died. This is a mortality of 1 in $5\frac{1}{2}$ for all ages and sexes even with the selection that was made. (Civiale, Traité de l'Aff. Calc. p. 552.)

3. In Bohemia, of 106 cases of calculus admitted into the hospitals, 60, the majority, were rejected as unfit for operation; only 46 were cut, of whom 38 recovered and 5 died; which gives a mortality in the ratio of 1 in 9 nearly, a very favourable result, and undoubtedly connected with the youth of the great majority of the subjects chosen. Of the 60 who were rejected, 28 were known to have died when the report was made. (Civiale, op. cit. p. 561.)

4. In the Lombardo-Venetian states, of 1104 patients affected with calculus who entered the public hospitals, 1044 were selected as fit for the operation, and 60 were rejected as unfit. Of the number cut 819 recovered and 217 died, which is a mortality of about 1 in $4\frac{3}{4}$ths for all ages and sexes. (Civiale, op. cit. p. 569.)

5. At the Hôtel Dieu of Paris from 1808 to 1830, 284 patients labouring under stone were admitted. Of this number only 100 were held fit to undergo the operation; and of them 56 recovered and 28 died; the mortality was therefore 1 in $3\frac{4}{7}$ths for all ages and sexes, in spite of the selection made. Of the 184 who were left to their fate, 133 were known to have died. What makes the extent of the mortality the more remarkable here, is the fact that of the 100 selected for operation 46 were children, 43 adults and only 11 aged; 24 or 25 of the deaths must have occurred among the adults and aged, so that the mortality among them could not have fallen far short of 1 in 2, a result which justifies M. Richerand's estimate of the consequences of lithotomy. (Civiale, op. cit. p. 595.)

6. "Dupuytren in the work on lithotomy which was published after his death has given what must be held as a very valuable and trustworthy document, in the shape of a table, of the results of 356 operations for stone done in Paris and the neighbourhood by different surgeons during a period of ten years, of the whole of which he had kept a register himself. The results with reference to the males, 312 in number, are at once appreciated by the figures of the following Table:—

| Ages. | Number of Cases. | Cured. | Died. | Ratio of Mortality. |
|---|---|---|---|---|
| From 3 to 15 | 97 | 88 | 9 | 1 in 11 nearly |
| — 15 to 30 | 59 | 51 | 8 | 1 in $7\frac{3}{8}$ |
| — 30 to 50 | 45 | 35 | 10 | 1 in $4\frac{1}{2}$ |
| — 50 to 70 | 74 | 36 | 18 | 1 in $4\frac{1}{9}$ |
| — 70 to 90 | 37 | 26 | 11 | 1 in $3\frac{4}{11}$ |
| Total...... | 312 | 236 | 56 | 1 in from 5 to 6 |

The mortality is not quite so favourable here as usual for subjects under the age of puberty; under 30, however, it amounts to 1 in $9\frac{1}{6}$ very nearly; but above 30 it is more than doubled, being as high as 1 in 4 exactly." And Dupuytren concludes that the general mortality of lithotomy is 1 in 5 or 6.

7. Cheselden has given us the particulars of his 213 operations.

| Ages. | Number of Cases. | Recovered. | Died. | Ratio of Mortality. |
|---|---|---|---|---|
| Under 10 | 105 | 102 | 3 | 1 in 35 |
| 11 to 20 | 62 | 58 | 4 | 1 — $15\frac{1}{2}$ |
| 21 — 30 | 12 | 9 | 3 | 1 — 4 |
| 31 — 40 | 10 | 8 | 2 | 1 — 5 |
| 41 — 50 | 10 | 8 | 2 | 1 — 5 |
| 51 — 60 | 7 | 3 | 4 | 1 — $1\frac{3}{4}$ |
| 61 — 70 | 5 | 4 | 1 | 1 — 5 |
| 71 — 80 | 2 | 1 | 1 | 1 — 2 |
| Total.. | 213 | 193 | 20 | 1 in $10\frac{13}{20}$ |

There are several other tables, no doubt accurate and trust-worthy, but the preceding are the main ones. Dr. Willis sums up:—"In estimating the risks from lithotomy, it is obvious, from the whole of the above statements, that AGE is an essential element in the reckoning. The operation, previous to puberty, is a very different affair from what it is after this great epoch in the life of man. Very little information is, in fact to be gained from statements of the results of the operation for all ages and sexes. It would seem generally to be of little moment who was the surgeon and what the operation: if the majority of subjects be youthful, the success will be considerable,—the mortality may be no more than 1 in 20 or 25; if, on the contrary, the majority of the patients be adult and aged, the mortality will certainly be from 1 in 5 to 1 in 2, according to the period of life." Dr. Willis indeed takes a gloomy view of the operation. For, independently of the usual dangers and disasters, he cites, as of far more consequence, *that* the presence of a stone for a term of years in the bladder is followed in numerous instances by an amount of disturbance in the function, and then of alteration in the structure, of the kidneys that as certainly occasions death as a succession of softening and suppurating tubercles in the lungs. The operation here but fires the mine at once, that would have exploded of itself before long. In other cases, again, the operation is beyond all question the immediate cause of the train of morbid phenomena, having the same general character and tendency, that so frequently follow it.

Dr. Willis having proved, what unfortunately no one can deny, the great fatality of lithotrity and lithotomy, and, certainly, not having underrated either, proceeds to develop his own operation, the agreeable tints of his picture of *that*, contrasting remarkably with the sombre hues in which he had painted *those*.

Lithectasy, or Cystectasy, the names he gives to the procedure, is founded on the dilatability of the membranous and prostatic part of the urethra, and is a sort of modification of the old Marian method. That operation was by incision of the external parts, and rapid dilatation or laceration of the internal, effected by a succession of blunt instruments called gorgets of different sizes. These instruments were introduced in pairs, through the membranous part of the urethra into the neck of the bladder, and being then separated from one another, they were made to distend and to tear. The first pair of gorgets having done their office, one of them was withdrawn, and upon the groove or tongue of that which still remained in the bladder, a second and larger-sized instrument was passed, then a third, and so on, until the amount of opening necessary for the passage of the clumsy tongs in use at the time, and the removal of these with the stone between their chaps, was obtained.

But it seems to be more particularly on a case of Sir Astley Cooper's that Dr. Willis leans. The subject of this case was a gentleman of middle age, who had some nine months previously undergone the usual operation for stone, in the course of which the rectum had been wounded, so that a fistulous opening remained between the bladder and the bowel, fæces passing with the urine and urine escaping with the fæces. In this state, and still suffering much from pain and irritability of bladder, the patient placed himself under the joint care of Drs. Neil and James Arnott, and Sir Astley Cooper. With a view to cure the fistulous communication between the urethra and rectum, Sir Astley Cooper made an opening into the urethra from the perineum, by which he passed a female catheter into the bladder, and immediately struck a stone. As it was likely to be small, Sir Astley did not object to the proposal now made by the Drs. Arnott, to try the effect of the new dilator in opening the passage for its removal. This instrument was accordingly used, and in the course of thirty hours the passage in the perineum, the membranous portion of the urethra and the neck of the bladder, were opened up till they were about two inches and a quarter in circumference, or three-quarters of an inch in diameter. The lithotomy forceps was then introduced by Sir Astley Cooper into the bladder, and the stone immediately felt, seized, and extracted. It was as large as a middling-sized walnut.

This operation was eminently successful. During the process of dilatation the patient had an uneasy feeling of distention, but nothing approaching to pain. In four days all the irritation of the bladder had subsided; the patient was able to retain his urine, and left his bed-chamber. On the ninth day the wound in the perineum was whole, and he began to take exercise abroad. And now for Lithectasy.

"Let the urethra be opened upon a grooved staff in the mesial line and behind the bulb to the extent of a few lines, as used to be done in the old operation of boutonnière, or as Cheselden did in his last operation." "To make such an opening, a lancet, in the absence of an implement with greater fixedness of parts, would perfectly suffice; and the urethra once open, we can dilate to the extent we please if we will but be patient. The vegetable tents of M. Guerin can always be procured, and nicely made would answer indifferently well to effect the needful opening. But an apparatus that is far preferable, that can be controlled to a nicety in its effects, and that is much more powerful than the tent, whilst its operation is still of the best kind, is the fluid-pressure dilator of Dr. Arnott. This is an instrument which consists of a cylindrical tube of silk lined with the thin gut of a small animal, to make it water tight, fixed upon a canula which traverses its axis, and having attached to its outer end a syringe guarded with a stop-cock, by which it may be forcibly distended with water or mucilage. This instrument when empty exceeds but little in size the bulk of the canula or catheter with which it is connected, and is therefore easily introduced along the groove of the staff into the bladder. When there it can be gradually distended to its utmost limits, and being made of so tough a material as silk, it expands evenly and is not liable to be burst by any degree of force that can be usefully employed. In Dr. Arnott's original case, 30 hours, during which the urine passed regularly guttatim by the canula, and the patient complained of no pain, but merely of a sense of distention, sufficed to accomplish dilatation to the extent necessary for the introduction of the forceps and the easy extraction of a stone having the dimensions of a walnut. And this without risk for one moment to the patient! The wound in the perinæum is a scratch; in itself and immediately it is hardly so dangerous as the puncture that is made in venesection; ulteriorly it can by no possibility be followed by any evil or unpleasant consequence; the urine never even comes into contact with the raw surface,—the dilator is introduced, and immediately this fluid, so poisonous to open wounds, finds no passage save through the canula; before the dilator is again withdrawn, the fresh surface has become covered with coagulable lymph through which nothing can penetrate; and the stone being removed, the gap might be closed with a couple of points of twisted suture and the urine made to come away at once by the whole length of the natural passage." 167.

Dr. Willis, armed with this operation, protests that stone in the bladder has lost a great proportion of its terrors. He does not seem to care for it the snap of a finger. It must be owned that this is looking at an almost untried operation rather sanguinely. That seems to be Dr. Willis's temperament. Our own blood, we must confess, runs a little more sluggishly in our veins, dulled, it may be, by many Winters, and by experience of the fallacy of human expectations. Nor does "the ungentle craft" present to us projectors or their plans altogether couleur de rose. Repeated disappointments, if they do not sour the critic's disposition, damp, at all events, his faith, and he lapses into the ungracious sceptic. Such is the tenure of his office, and, alas! by that tenure we hold ours.

When trials have confirmed Dr. Willis's anticipations, when it is proved that the neck of the bladder, which often will not bear a bougie or sound, will bear such dilatation as this method implies, when the ordinary risks that wait on all meddling with an irritable and irritated viscus, are found to dissolve away before Lithectasy, then we will join our voice with Dr. Willis, and proclaim that stone *has* lost its terrors. But till then we must pause, and though we cannot but admire the lively talent, the enthusiasm, and the hearty earnestness of Dr. Willis, we regret that our own temper is more sober and more sad. We hope sincerely, for the sake of humanity, that he is right and we are wrong.

